# Seven years of pediatric robotic-assisted surgery: insights from 105 procedures

**DOI:** 10.1007/s11701-025-02257-w

**Published:** 2025-04-15

**Authors:** Ciro Esposito, Lorenzo Masieri, Claudia Di Mento, Mariapina Cerulo, Fulvia Del Conte, Vincenzo Coppola, Giorgia Esposito, Francesco Tedesco, Annalisa Chiodi, Francesca Carraturo, Roberta Guglielmini, Francesca Alicchio, Micaela Borrelli, Leonardo Continisio, Maria Escolino

**Affiliations:** 1 Pediatric Surgery Unit, Federico II University Naples, Via Pansini, 5 80131 Naples, Italy; 2 Urology Unit, Meyer University Hospital Florence, Florence, Italy; 3 Internal Medicine Unit, Federico II University Naples, Naples, Italy; 4 Pediatric Surgery Unit, Ruggi D’Aragona Hospital, Salerno, Italy; 5 Department of Molecular Medicine and Medical Biotechnologies, University of Naples, Naples, Italy

**Keywords:** Robotic-assisted surgery, Pediatrics, Pediatric urology, Robot, Learning curves

## Abstract

Robotic-assisted surgery (RAS) has recently expanded its role in pediatric patients. We conducted a retrospective review of 105 cases over 7 years (2017–2024) to evaluate outcomes, efficiency, and training experiences. A total of 105 children (58 boys, 47 girls) aged 2–15 years underwent robotic-assisted procedures using the Da Vinci Xi system. The most common indications were ureteropelvic junction obstruction (*n* = 33), varicocele (*n* = 29), and primary obstructive megaureter (*n* = 16). Two senior surgeons performed the procedures, training seven junior surgeons via the dual-console system. Statistical analysis included paired t-tests for docking time and operative duration comparisons, and Fisher’s exact test for categorical variables. Docking time significantly improved over time from 45 to 15 min (median 25 min) (*p* = 0.001). The total operative time significantly decreased over time (p = 0.001), with a median of 125 min (range 50–250). Robotic system-related issues were reported in 3/105 (2.8%). Conversion to laparoscopy was necessary in 1 (0.9%). Postoperative complications (Clavien grade 3b) occurred in 2/105 (1.8%) patients, requiring reintervention. The median hospital stay was 2 days (range 1–7). Monthly case volume increased from 1–2 to 4–7. Our 7 year experience with pediatric RAS demonstrates its safety, effectiveness, and growing role, especially in pediatric urology. It offers ergonomic advantages and facilitates training but is still limited by cost, larger instrument size (8 mm), and longer setup times compared to laparoscopy. Future developments, such as smaller robotic instruments and single-port technology, may help overcome these limitations and expand the applicability of RAS to younger and smaller patients.

## Introduction

Over the past 30 years, minimally invasive surgery (MIS) has become widely adopted in pediatric surgery. MIS offers several advantages over open surgery, including reduced postoperative pain, shorter hospital stays, faster recovery times, and improved cosmetic outcomes. The development of robotic-assisted surgery (RAS) has significantly advanced the concept of minimally invasive procedures [1–7].

RAS provides several benefits, such as superior 3D visualization, enhanced surgeon ergonomics, elimination of hand tremors during prolonged procedures, and improved instrument dexterity—especially for complex reconstructive surgeries [1, 3, 4, 6]. In addition, RAS has a shorter learning curve for trainees, facilitating faster skill acquisition [8, 9]. According to international literature, the primary fields of application for RAS include urology, general surgery, and gynecology [3, 4, 10–12]. However, most scientific evidence is based on studies conducted in adults. While RAS has been extensively studied in adult populations, there is limited comprehensive analysis of its application in pediatric surgery.

Following 3 decades of experience in pediatric MIS—beginning in the early 1990s—we launched a pediatric robotic surgery program in late 2017 [13, 14].

After 7 years of experience and more than 100 robotic-assisted surgeries performed in pediatric patients, we conducted a retrospective review of 105 consecutive cases treated at our center during this period. This study aimed to analyze the lessons learned from our experience and, more importantly, to share insights on RAS, drawing from 30 years of experience with laparoscopy and 7 years with robotic surgery.

## Patients and methods

### Study design

We conducted a retrospective cohort study that included all patients who underwent RAS in our pediatric surgery division between November 2017 and November 2024. Patients older than 1 year and weighing more than 15 kg were selected for the robotic approach. Exclusion criteria included a weight of less than 15 kg, comorbidities such as hemodynamic instability, pulmonary hypoplasia, or other general conditions that contraindicated pneumoperitoneum based on the anesthesiologists’ evaluation, as well as cases where patients or parents/caregivers opted against the robotic approach.

The patient cohort consisted of 58 boys and 47 girls, with median age of 7 years (range 2–15) and median weight of 25 kg (range 19–71). In our series, we identified 12 different indications for RAS: 33 cases of ureteropelvic junction obstruction (UPJO), 9 cases of vesicoureteral reflux (VUR), 29 cases of varicocele, 7 ovarian tumors, 16 cases of primary obstructive megaureter (POM), 3 gallbladder diseases, 2 renal tumors, 2 cases of appendicitis, 1 bilateral inguinal hernia, 1 case of gastroesophageal reflux disease (GERD), 1 renal stone and 1 bladder stone.

This retrospective cohort study was approved by the Institute Review Board (IRB) of Federico II University of Naples, Italy (approval number 2024/Z953).

Study population is summarized in Table 1.

**Table 1:** Patients’ demographics

Patient cohort n		Median age Years (range)	Median weight kg (range)	Indication for RAS	
				Number	Type
Total	105	7 (2–15)	25 (19–71)	33	UPJO
Male	58			9	VUR
Female	47			7	Ovarian tumor
				29	Varicocele
				16	POM
				3	Gallbladder disease
				2	Renal tumor
				2	Appendicitis
				1	Bilateral inguinal hernia
				1	GERD
				1	Renal stone
				1	Bladder stone

*RAS* Robotic-assisted surgery, *UPJO* Ureteropelvic junction obstruction, *VUR* Vesicoureteral reflux, *POM* Primaries obstructive megaureters (POM), *GERD* Gastroesophageal reflux disease

### Operative techniques

The Da Vinci Xi Surgical System with a double console system (Intuitive Surgical Inc., Sunnyvale, CA, USA) was utilized for RAS, as it was the only robotic platform available at our university hospital.

We employed a setup consisting of three 8-mm robotic port and one 5-mm laparoscopic port for the bedside assistant. All robotic procedures were performed by two independent senior surgeons with over 15 years of experience in MIS, along with three junior surgeons under their mentorship. This was made possible by utilizing the two different robotic console systems available in the operating room. Regarding the training protocol for junior surgeons, they typically begin with at least 30 h of simulation sessions using the Da Vinci Xi Robotic simulator. Following this, they attend wet lab courses with animal models for a total of 10 h before progressing to in vivo surgery.

All procedures were performed under general anesthesia with orotracheal intubation. Among the 105 procedures, 33 were performed on patients with UPJO, including 28 primary surgeries and 5 redo procedures following previous open pyeloplasty. In the 28 primary cases, an Anderson–Hynes pyeloplasty was performed, while 3 out of the 5 redo cases underwent Anderson–Hynes pyeloplasty, and 2 required a uretero-calycostomy. For the 30 patients with left varicocele, we performed a Palomo lymphatic-sparing procedure in all cases, utilizing ICG fluorescence technology with the Firefly^®^ system.

Among the nine patients with VUR, five had unilateral pathology, and four had bilateral pathology. In all cases, we performed a Lich–Gregoir procedure with extravesical ureteral reimplantation.

In the seven cases of ovarian tumors, a salpingo-oophorectomy was performed using ICG fluorescence technology and robotic vessel sealer device. The ovary was placed into a retrieval bag and removed through the umbilical incision. In one patient with a 20 cm ovarian mass, the tumor was isolated robotically and subsequently removed via a Pfannenstiel incision.

In the 16 cases of POM, we performed a resection of the stenotic segment of the ureter followed by extravesical reimplantation using the Lich–Gregoir technique, combined with ureteral tapering.

For the three patients with gallbladder stones, a robotic cholecystectomy was performed with the assistance of ICG fluorescence technology.

In cases involving renal tumors, a robotic nephrectomy was performed, complemented by ICG fluorescence guidance and lymph node sampling.

In the two cases of appendicitis, a robotic appendectomy was completed using the double endoloop technique. For the patient with bilateral inguinal hernia, robotic repair was conducted using the purse-string suture technique, as described by Montupet.

For the renal stone located in the renal pelvis, a pyelotomy was performed, the stone was extracted using an endobag, and a double-J stent was placed. In the case of 3 cm bladder stone in a neurologically impaired patient with neurogenic bladder, the stone was removed through a median cystotomy, placed in an endobag, and the bladder was closed in two layers.

Finally, in the single case of gastroesophageal reflux disease (GERD), the procedure required conversion to laparoscopy due to an enlarged liver obstructing the view of the esophageal hiatus. To address this, two additional trocars were placed, and a Nathanson hepatic retractor was used to elevate the liver, allowing for adequate visualization and completion of the procedure.

### Data analysis

All quantitative variables were expressed as median and range. For comparisons of docking time reduction over the years, a paired t-test was applied to evaluate statistical significance between early and late periods of the study. Categorical variables, such as the proportion of robotic technical issues and conversion rates, were analyzed using the Chi-square test or Fisher’s exact test.

All statistical analyses were performed using SPSS Statistics (IBM Corp., Armonk, NY, USA), version 23.0. A p value < 0.05 was considered statistically significant.

## Results

During the first 6 years of our pediatric robotic program, we operated on 1–2 patients per month, with an increase to 4–7 patients per month just in the last year. The primary indication for RAS in our series was urological procedures, accounting for 59 out of 105 patients (56%). The docking time significantly improved over the study period, decreasing from 45 to 15 min (median 25 min). Statistical analysis using a paired t-test confirmed this reduction was statistically significant (*p* = 0.001), demonstrating a consistent learning curve improvement among the surgical team.

The total operative time ranged from 50 to 250 min, with a median of 125 min. A paired t-test comparing early versus late cases in the study confirmed a statistically significant reduction in operative time (*p* = 0.001), demonstrating improved efficiency with the robotic system and enhanced proficiency of the surgical team.

All procedures, except one, were successfully completed using RAS. In a single case involving a girl with gastroesophageal reflux disease (GERD), conversion to laparoscopy was required due to challenges in adequately retracting the liver and obtaining a clear view of the esophageal hiatus.

Of the 105 robotic-assisted procedures, 3 cases (2.8%) experienced technical issues related to the robotic system. Two cases involved instrument malfunctions, specifically with the robotic scissors, which required immediate replacement before proceeding with the surgery. One case encountered a brief delay in instrument response, with a few seconds between joystick input and instrument movement. This issue was immediately addressed and resolved with real-time technical support from Intuitive Surgical. None of these technical issues resulted in conversion to laparoscopy or open surgery.

Postoperative surgical complications occurred in 2 out of 105 patients (1.8%). The first case involved a 15-year-old boy who underwent robotic varicocelectomy and developed on second postoperative day hemoperitoneum secondary to trocar site bleeding. He required reoperation to evacuate the hemoperitoneum and control bleeding from a parietal vessel (Clavien grade 3b). The second complication occurred in a 5-year-old boy who underwent robotic-assisted pyeloplasty. He developed a perirenal collection due to the dislodgement of the double-J stent, which migrated from the bladder into the ureter. Ureteroscopy was successfully performed to retrieve the displaced stent and replace it with a larger one, followed by laparoscopy to drain the perirenal collection (Clavien grade 3b).

The median hospital stay was 2 days (range 1–7). We identified several factors that influenced the variation in hospital stays. Shorter stays (1–2 days) were observed in patients undergoing varicocele repair, inguinal hernia repair, and appendectomy, as these procedures had lower postoperative recovery demands. Longer stays (5–7 days) were necessary for patients undergoing nephrectomy or complex ureteral reconstructions, due to the need for extended postoperative monitoring, pain management, and, in some cases, temporary urinary stents or drains or in those developing surgical complications and needing reoperation. Postoperative factors, such as the need for intravenous antibiotic therapy or the presence of surgical drains, also contributed to prolonged hospitalization.

Results are summarized in Table 2.

**Table 2:** Summary of study’s results

Median docking time, min (range)	25 (15–45)
Median operative time, min (range)	125 (50–250)
Conversion rate, n (%)	1/105 (0.9%)
Technical issues with the robotic system, n (%)	3/105 (2.8%)
Postoperative complications, n (%)	2/105 (1.8%) (Clavien grade 3b)
Median hospitalization, days (range)	2 (1–7)

## Discussion

Our study presents a comprehensive evaluation of our 7-year experience with pediatric robotic-assisted surgery (RAS), highlighting both its clinical implications and operational challenges. With an experienced surgical team and a structured training program, our findings provide valuable insights into the evolving role of RAS in pediatric procedures. Our results support the broader adoption of RAS in pediatric surgical practice. The main advantages of RAS over traditional MIS include enhanced precision, shorter learning curves and improved ergonomics, making it particularly suitable for complex pediatric reconstructive procedures. Many urological procedures, such as pyeloplasty [15–17, 19], bladder reimplantation [20–23], and dismembered reimplantation with ureteral tailoring [24–26], are technically demanding and require long operative times. These factors make laparoscopic execution of such procedures particularly challenging, primarily due to suboptimal ergonomics for the surgeon and difficulty in intracorporeal suturing.

A critical limitation of RAS is its reduced applicability to patients under 15 kg, primarily related to the current robotic instrument size (8 mm), the risk of port clashing and slippage, and the challenges posed by smaller anatomical structures. While some studies have reported neonatal cases performed using the Da Vinci Xi platform [27, 28], our preference for laparoscopy in patients under 10–15 kg is based on the superior maneuverability and feasibility it offers in this population. Alternative strategies include advanced laparoscopic techniques, the development of smaller robotic instruments, and potential advancements in single-port (SP) robotic systems designed for pediatric applications.

The primary indication for RAS in pediatric patients was represented by urological conditions, in accordance with the previous literature [3, 4, 10, 14]. Complex reconstructive procedures such as pyeloplasty or bladder reconstruction, especially in patients with difficult anatomic conditions, are the most common robotic-assisted procedures described in pediatric age [15–18].

Our findings align with existing literature on pediatric RAS, demonstrating similar trends in learning curve progression and efficiency gains. The median docking time in our study decreased to from 45 to 15 min, consistent with previously reported improvements in robotic proficiency over time [29–31]. Similarly, Ashraf et al. reported a reduction in mean docking time from 23 to 15 min after 30 cases, indicating that robotic efficiency improves significantly with surgeon and team familiarity [32]. Our 1.8% postoperative complication rate compares favorably to reported rates in other pediatric RAS studies, which range from 2 to 5% [31]. Despite concerns about the cost-effectiveness of robotic surgery, our findings reinforce the evidence that RAS can yield long-term benefits [29–31]. The reduction in hospital stays and quicker recovery times may offset the initial financial investment required for acquisition of robotic systems.

Our experience highlights the importance of a skilled laparoscopic team to effectively operate the transition from laparoscopy to robotics and integrate both techniques within the same surgical unit. In pediatric RAS, teamwork is even more critical than in adult RAS, with each team member playing a vital role in the surgical process. Nurses are essential for docking the robotic system, assisting at the operative table, configuring the robotic equipment, and managing instrument changes. In addition, pediatric RAS typically benefits from the presence of two anesthetists to ensure precise and delicate management of pediatric anesthesia [2]. In our operating room, we use the Da Vinci Xi robotic system, which is equipped with four arms. This setup enables the console surgeon to operate with four instruments simultaneously. However, most surgeons, including our team, prefer using three 8-mm arms, supplemented by an additional 5-mm laparoscopic trocar for the bedside surgeon. The bedside surgeon plays a crucial role in robotic procedures, assisting the console surgeon by retracting anatomical structures and, more importantly, by introducing and removing needles and cutting sutures. However, the bedside surgeon faces certain challenges. They must operate within a confined space between the robotic arms, which can be difficult. In addition, their view of the operative field is limited, as they rely on a small screen positioned between the robotic arms, whereas the console surgeon benefits from a high-definition 3D view.

The Da Vinci Xi platform offers several significant advantages over traditional laparoscopy. One key benefit is the ability to use monopolar and bipolar coagulation simultaneously on two instruments, typically with scissors configured for monopolar energy and Maryland forceps for bipolar energy. The quality of both monopolar and bipolar energy is outstanding and significantly superior to what is available in laparoscopy. In addition, the Xi platform features two highly efficient energy devices: the Vessel Sealer Extend providing two-step seals and cuts and the SynchroSeal, providing single-step sealing and transection. Moreover, the integrated software Firefly® enables the use of indocyanine green (ICG) near-infrared fluorescence (NIRF) technology, providing real-time tissue visualization and improving surgical outcomes [33].

Another significant advantage of the Intuitive platform over laparoscopy is its ability to record comprehensive surgical data. This includes details such as procedure duration, instrument usage time, docking time, and other critical metrics. These features are particularly valuable for surgeons, facilitating detailed analysis and optimization of surgical performance and outcomes.

Training the next generation of surgeons is a crucial component of our robotic program. This system significantly enhances the training process [8], allowing junior surgeons to operate from a second console, where they can perform parts of the procedure independently while being closely supervised by an expert surgeon at the main robotic console. This setup provides hands-on experience while maintaining a high level of oversight and guidance.

As previously mentioned, our robotic-assisted experience is exclusively with the Da Vinci Xi robotic system. One significant limitation of this system is the size of ports (8-mm), which are too large for infants weighing less than 15 kg. The new Intuitive single-port (SP) platform might offer a valuable solution to perform RAS in smaller patients [34–36]. To further optimize pediatric RAS, future research should focus on the feasibility of single-port robotic systems in pediatric patients, which may overcome current size limitations. Although the Intuitive SP platform is currently only approved for adult use, future modifications could make it a viable option for younger patients. The integration of artificial intelligence (AI)-driven robotic assistance presents a promising avenue for improving precision and shortening learning curves in pediatric RAS. AI can aid in intraoperative decision-making, instrument control, and surgical planning. Long-term studies comparing postoperative outcomes between RAS, traditional MIS and open surgery will be essential for validating the durability of robotic-assisted procedures in terms of functional and cosmetic results.

One of the major strengths of this study is the presence of two highly experienced surgeons leading the RAS program. Their expertise contributed to improved surgical efficiency and facilitated structured training for junior surgeons. However, the logistic challenges of a general hospital remain a limiting factor. Our centralized robotic operating room (OR) is shared with adult surgical disciplines and located approximately 750 m away from our pediatric surgery unit. This necessitates patient transport via ambulance, introducing delays and inefficiencies. The competition for access to the robotic OR also limits the number of pediatric procedures performed, with case volumes increasing only in the final year of the study. Institutional financial constraints may also limit the scalability of RAS programs in pediatric surgery. While the benefits of RAS are well documented, the high initial investment in robotic systems remains a hurdle for many hospitals. Future strategies for overcoming these limitations should explore solutions such as establishing dedicated pediatric robotic ORs to enhance scheduling efficiency, implementing institutional investment in robotic technology and collaborative partnerships with industry stakeholders to reduce costs, and developing smaller and more affordable robotic platforms tailored to pediatric surgical needs.

In conclusion, our 7 year experience with pediatric robotic-assisted surgery (RAS) demonstrates its safety, effectiveness, and growing role in pediatric surgical practice, especially in pediatric urology. The significant reduction in docking and operative times, along with a low rate of complications and conversions, highlight the efficiency and reliability of the robotic platform. These findings reinforce the advantages of RAS, including superior precision, improved surgeon ergonomics, and a structured training pathway for junior surgeons through the dual-console system.

While RAS offers distinct benefits over conventional MIS, its widespread adoption in pediatric surgery is still limited by high costs, larger instrument size (8 mm vs standard 3 mm laparoscopic instruments), and longer setup times. Future developments, such as smaller robotic instruments and single-port technology, may help overcome these limitations and expand the applicability of RAS to younger and smaller patients.

Given its advantages, RAS is likely to play an increasingly central role in pediatric urological procedures. However, further multicenter studies and long-term outcome evaluations are necessary to determine its full impact on clinical practice and cost-effectiveness compared to traditional MIS.

## Data Availability

No datasets were generated or analyzed during the current study.
